# Immersive 360° videos in health and social care education: a scoping review

**DOI:** 10.1186/s12909-021-03013-y

**Published:** 2021-11-24

**Authors:** Carolyn Blair, Colm Walsh, Paul Best

**Affiliations:** School of Social Sciences, Education and Social Work, Queen’s University Belfast, 6 College Park, Belfast, Northern Ireland

**Keywords:** 360° videoing, 360° videos, Head mounted display, Health and social care education, Immersive technology, Literature review, Pedagogy, Training, Virtual reality, VR

## Abstract

**Background:**

Research on the pedagogical use of immersive 360° videos is a rapidly expanding area within health and social care education. Despite this interest, there is a paucity of empirical data on its application.

**Method:**

A scoping review methodology framework was used to search for relevant articles published between 1970 and July 2021. Six databases were used to identify studies using immersive 360° videos for training and education purposes within health and social care: PubMed, Ovid Medline, Psych Info, Psych Articles, Cochrane Database and Embase. Research questions included: Is there any evidence that immersive 360° videos increase learning outcomes and motivation to learn in health and social care education? What are the key pedagogical concepts and theories that inform this area of research? What are the limitations of using immersive 360° videos within health and social education? The four dimensions contained within Keller’s ARCS model (attention, relevance, confidence and satisfaction) frame the results section.

**Results:**

Fourteen studies met our inclusion criteria. Learning outcomes confirm that immersive 360° videos as a pedagogical tool: increases attention, has relevance in skill enhancement, confidence in usability and user satisfaction. In particular, immersive 360° videos has a positive effect on the user’s emotional response to the learning climate, which has a significant effect on users’ motivation to learn. There was a notable lack of pedagogical theory within the studies retrieved and a general lack of clarity on learning outcomes.

**Conclusion:**

Studies examining the effectiveness of such interventions remains weak due to smaller sample sizes, lack of randomised control trials, and a gap in reporting intervention qualities and outcomes. Nevertheless, 360° immersive video is a viable alternative to VR and regular video, it is cost-effective, and although more robust research is necessary, learning outcomes are promising.

**Future directions:**

Future research would do well to focus on interactivity and application of pedagogical theory within immersive 360° videos experiences. We argue that more and higher quality research studies, beyond the scope of medical education, are needed to explore the acceptability and effective implementation of this technology.

## Background

Educating and training students to work in health and social care settings is both complex and nuanced. Yet with chronic underfunding in areas, such as staff development and retention, mounting staff shortages [1] and the continuing fallout from the Covid-19 pandemic, it has never been more important to ensure a healthy pipeline of well prepared and trained health and social care staff. The Commission on the future of the NHS after COVID-19 [1] state that more proportional training funds must be allocated to create enhanced opportunities and all workers should have access to opportunities for education, career progression, and professional development. It’s evident that failure to invest in the appropriate training of staff have clear implications, namely, staff attrition is likely to be higher and outcomes for service users more likely to worsen [2]. The scarcity of training and professional development for some staff groups is noteworthy, for example only 5% of the Health Education England budget is currently allocated to training clinical and non-clinical support staff [3]. Furthermore, due to the COVID-19 pandemic and the continuing measures to control infection rates, those in health and social care education have seen a dramatic decline in their face-to-face exposure to all aspects of their training creating additional barriers for educators [4]. Consequentially, there are renewed calls for innovative, solution focused thinking in order to maximise the potential offered to us by new and emerging technologies. As training in certain health and social care settings has been restricted, educators have to be responsive and adaptive in preparing professionals and students for ‘real world’ practice [5–7]. As such, the need for and interest in high-fidelity, simulation-based learning has significantly increased [8–10]. One group of technologies that are starting to show promise in the area of health and social care education are ‘Immersive technologies’. Immersive technology is defined as a set of interfaces, applications, and software that create augmented simulations for experiences and perfect interactions between human beings and technology [11]. These include Virtual Reality (VR), Augmented Reality (AR), Mixed Reality (MR) and 360° videos. VR and AR are two of the most popular technologies used within this space however production costs are higher therefore issues remain regarding their accessibility within educational settings. Although hardware costs and requirements have steadily declined over the years [12], a recurring challenge is related to the costs and complexity involved in the production of highly realistic and customisable environments. VR and AR either require a high level of programming skills and effort or significant financial means to outsource these efforts [13]. MR is a new pedagogy that involves both physical and virtual elements including both learning content and making use of effective tools for realisation [14]. 360° videos consist of video recordings, made with a device able to simultaneously capture and combine scenes in a 360° degrees perspective [15]. Unlike computer-generated, 3D graphics-based VR, 360° videos provide a more affordable method for rapidly creating VR environments in which users can experience a sense of immersion [16].

The focus of this review is on the use of immersive 360° videos in health and social care education. Immersive 360° videos are a low-cost alternative to VR, using a 360° camera to capture the environment (point and click). It has also shown equal promise in achieving ‘spatial presence’ (i.e. the extent to which the immersive environment feels real) [17]. A key difference between immersive 360° videos and VR, is the former’s focus on ‘real’ rather than ‘simulated’ (virtual) imagery [18]. Immersive 360° videos with head mounted displays (HMDs) enables the viewer to ‘immerse’ themselves within a user-controlled training simulation or setting, whereby one feels as if they are ‘present’ in that space [19]. When viewing immersive 360° video content, users are not restricted to a single point of view as specialist cameras have enabled the simultaneous capture and recording of the entire environment from a fixed point. The viewer can control and change the viewing angle at any stage through head movement (if using an HMD) or through touch/mouse (if displayed on a screen). Immersive 360° videos share many characteristics with VR, including audio-visual productions with 360° × 180° navigation as well as the use of an HMD to view content. However, notably 360° videos are known to have a lack of interactivity when compared with VR. The primary focus of this review is on *‘immersive 360° videos’*, therefore we have defined this as 360° content viewed through a Virtual Reality (VR) headset or another HMD (as opposed to a computer screen). The benefits of including HMD’s include the ability to remove external (real world) stimuli (both visual/auditory). By utilising an HMD to experience a 360° videos, participants are fully immersed in the scene, able to see different fields of view similar to what someone would experience when moving their head to look at different directions in real life [20]. The benefits are evident however we are yet to see the widespread adoption of immersive 360° videos. This omission may be in part, due to the lack of robust research evidence demonstrating the ‘added value’ of this technology beyond traditional two-dimensional (2D) approaches (standard video). Although largely based on education based studies using VR, the advantages of immersive technology are based around the interactivity it provides. When using this technology the student has freedom of navigation, the ability to inquire into the properties of virtual objects and the capacity to access the specific information needed for learning therefore the student can obtain more powerful learning gains [21, 22]. Although progress is being made in exploring ways to make 360° videos more interactive, it must be noted that it is often limited in comparison to VR, the impact of this limitation is yet to be fully explored in health and social care education.

Immersive Technology has received extensive academic attention and a productive interest in educational studies, since their integration facilitates the task of teaching and improves learning experiences [23–27]. Although this is an emerging area, evidence to date shows it has the potential to provide requisite training for professionals working in health and social care [25, 26, 28]. Encouraged by innovations, such as affordable 360° cameras and HMDs, it is now more feasible than ever for educators and health professionals to develop bespoke 360° training scenarios and content [25, 29, 30]. The approach has significant utility given that implementation can occur as and when required, often at short notice. Given that ‘in-situ’ simulation offers the opportunity for development and a form of education that can create, not just translate, knowledge supported in a scaffolded, supportive environment [28]. The impact is potentially transformative, given the ability to place the learner in an authentic or real scenario in which to develop their knowledge, skills and role expectation [31–34]. However, there remains a lack regarding the most efficient ways to produce and display 360° content for health and social care training and pedagogy, those that do exist lack generalisability or sufficient theoretical depth [31–34]. 360° technology is relatively new; therefore, the evidence base is disparate, and to date, it is unclear how exactly it should be used as a useful pedagogical tool [35, 36]. The limited body of knowledge on the pedagogical use of immersive 360° videos indicates that more research is needed to explore acceptability and effectiveness of the technology [25–27, 32, 37, 38].

### Theoretical considerations

When asking what the added value of immersive 360° videos as a pedagogical resource for application in health and social care actually is, it is important to consider the theoretical underpinnings. In the case of immersive technologies, core concepts, such as *Presence* and *Immersion* have been exceptionally popular, particularly in relation to the user experience and emotional engagement [39, 40]. The sense of presence (SoP) is a subjective experience as the participants using the technology sense being immersed in the VR environment, as if they are “actually physically there,” perceiving virtual content as real [39]. Immersion is the objective description and reflects the extent to which technology allows engagement of the users to better represent reality, involving user’s panoramic view of the content during the experience removing the awareness of other physical realities present in the environment [40]. However, the efficacy of immersive environments as practical settings for learning is much less understood [38]. We believe that two theories are of significant importance when exploring the effectiveness of immersive environments Dale’s [41] ‘*Cone of Experience’* (CoE) and Keller’s [42–45] ARCS-V model of motivational learning design.

**Fig. 1:**
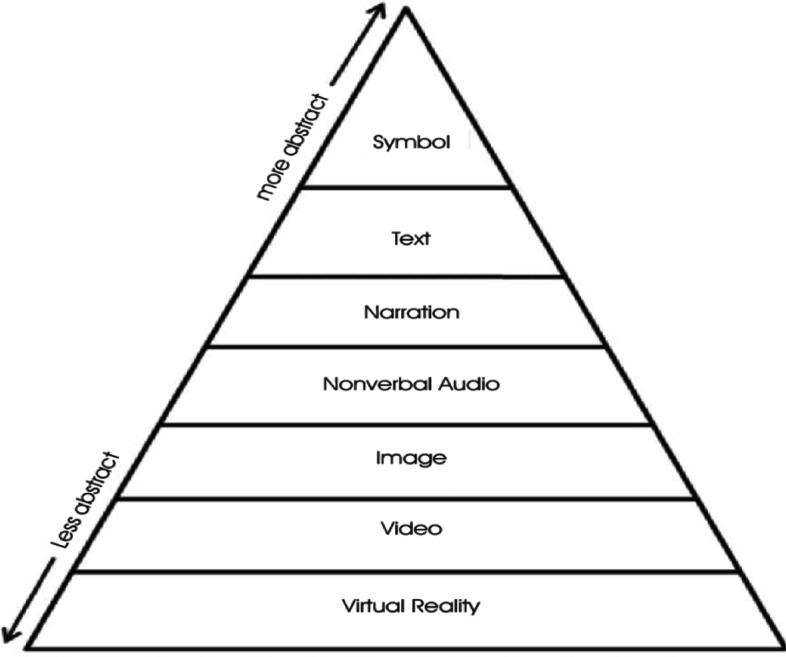
Multimedia Cone of Abstraction’ [17]

Dale’s [41] ‘*Cone of Experience’* (CoE) is a seminal theory on media-based instructional design. This theory posits the progression of learning based on levels of abstraction. He hypothesised that learning experiences became more embedded as learners engaged with others in applied settings. For example, Dale believed that direct and dramatised experiences were more concrete representations of reality than visual (reading) or verbal (listening) symbols. However, he was careful not to ascribe a hierarchy to these levels in recognition that different approaches may be better suited within different learning contexts and appeal to different learning styles. Building on the work of Dale [41]; Baukal, Ausburn and Ausburn [46] updated the CoE model and combined it with Mayer’s [47] Cognitive Theory of Multimedia learning to develop a new *‘Multimedia Cone of Abstraction’* theory (MCoA) (see Fig. 1). They suggested that while Dale’s [41] CoE model was useful, it pre-dated advances in modern multimedia technology, which needed to be accounted for. One such example was how the inherent features of VR technology, such as the interactive dynamic visuals and wider research, were advantageous over static visualisations for learning [48, 49]. In relation to immersive 360° videos, Baukal, Ausburn and Ausburn [46] note that there are two forms of VR – ‘real’ and ‘simulated’. The former (akin to 360° videos) is the least abstract version and “involves a user-controllable virtual reality simulation using actual images such as photographs of things like objects or scenes” (p.19). Simulated VR is also user-controllable, but uses “simulated graphics, such as computer-aided drawings, instead of actual photo-real images” (p.19). Both can be further enhanced by the introduction of narrative and text, bearing in mind potential issues with Cognitive Load [50]. In theory, immersive 360° videos may therefore appeal to a broad range of learners and therefore has the potential to be effective as a versatile training tool in health and social care education.

Beyond considering the use of technology to enhance, replicate or simulate settings/tasks for professional training, one must also consider the motivation of learners to engage with the learning task itself, therefore, theories regarding motivational learning are particularly relevant. This is particularly relevant as previous research has shown a high level of satisfaction and engagement when using immersive technologies [25–27]. Therefore a further theoretical consideration based on motivational learning design is specifically the ARCS-V (*attention, relevance, confidence, satisfaction and volition)* model [42–45]. Keller [44] proposes an interactionist approach to developing learning experiences, whereby thinking and behavioural based learning is motivationally influenced. Keller’s theory [42–45] on the motivation to learn is distinctive as it includes a focus on the importance of setting learning outcomes while also ensuring the learner’s needs are met. The five dimensions contained within Keller’s ARCS-V model [42–45] are viewed as critical for creating a motivational learning environment. *Attention* activities range from developing unexpected events to mentally simulating challenges that increase curiosity. *Relevance* is the extent to which the learner sees value in the activity. By developing activities wherein, the learner establishes ‘positive expectancies for success’ *confidence* is likely to increase. *Satisfaction* refers to the positive feelings gained from the learning activity and can also be gained from an opportunity to demonstrate learning. Later, a fifth category, *volition*, was added to create the ARCS-V model [45] which implies that once the educational environment is designed, the audience is more likely to feel motivated and will self-regulate their behaviour to achieve their goals. The validity, reliability and impact of the ARCS model [42–44] have been tested in several different contexts, such as technology-supported learning, e-learning, user-orientated design and health and social care pedagogy [43, 51–53]. The ARCS model [42–44] will frame the results sections in this review by way of uncovering what factors are facilitator and barriers of creating a motivational learning environment.

## Method

Given the fact that knowledge in this area is emergent, a scoping review was chosen as the appropriate method of synthesis [54, 55]. Methodologically, this study was guided by the Arksey and O′Malley’s [54] methodological framework predominant literature on conducting scoping reviews for data sourcing, collation and extraction. The number of studies identified and selected for inclusion were reported according to the PRISMA (Preferred Reporting Items for Systematic Reviews and Meta-Analyses) guidelines [56].

### Research questions

The review aimed to summarise, identify research gaps, and to make recommendations for the future research based on the following research questions:

1. Is there any evidence that immersive 360° videos increase learning outcomes and motivation to learn in health and social care education?
2. What are the key pedagogical concepts and theories that inform this area of research?
3. What are the limitations of using immersive 360° videos within health and social education?

### Identifying relevant studies

**Fig. 2:**
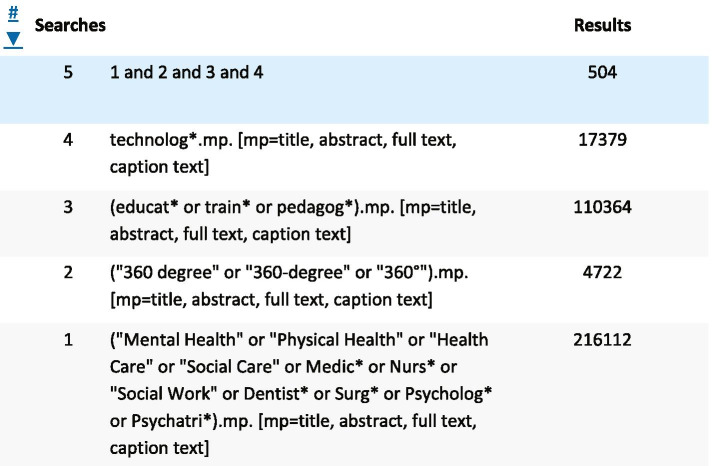
Example search string

We searched for relevant articles published between 1970 and July 2021 using six databases: PubMed (*n* = 456), Embase (*n* = 144), Ovid Medline (*n* = 68); Psych Info (*n* = 52); APA psych articles (*n* = 504), Cochrane Trials (*n* = 26) to identify studies using immersive 360° videos for training and pedagogy purposes within health and social care. For an example of the search string see Fig. 2.

Once we completed these electronic searches, we conducted a hand-search of Google Scholar and scanned the reference lists from relevant papers to identify other papers that may not have been found in the initial search.

### Study selection

Once we identified the relevant literature, our team established the exclusion and inclusion criteria to apply to the papers (see Table 1). For example, we excluded studies which did not use a Head-Mounted Display (HMD), and those which used Google Cardboard. We made the decision to exclude google cardboard given that we wanted to focus on higher quality HMDs. The added benefits of higher quality headsets including alternatives to google cardboard which use mobile phones (such as Samsung Gear) are adjustable lens, increased field of view and tracking head movement all of which permit a more immersive experience. By applying the exclusion criteria to the abstracts, we reduced the numbers of papers that needed to be read in detail to 84 papers. Given the relative infancy of immersive 360° videos, we included all published studies (including conference proceedings, providing there was a full text of the paper) and study design types that collected primary empirical data and reported pedagogical outcomes.

**Table 1:** Inclusion and exclusion criteria

Inclusion Criteria	Exclusion Criteria
Health and social care professionals or students	Patients or general public
Studies using a 360 degree camera	Virtual Reality/Augmented Reality/Mixed Reality focused or other non-relevant technology
Publications reporting on qualitative, quantitative or mixed method studies, conference proceedings (full text).	Literature reviews/systematic reviews/conference abstracts/any publication which does not provide essential detail (e.g. participant numbers)
Studies using head mounted displays (HMDs)	Google Cardboard for smartphone use.
Studies with education, training or pedagogy related outcomes	Studies which focused solely on the effectiveness of the equipment

The results were imported into the screening tool, Rayyan [57]. Rayyan is an online tool which allows for the management and screening of articles intended for inclusion in reviews. After removing duplicates, the titles and abstracts were independently screened by two reviewers (CB and PB). CB and PB then independently reviewed the full text of each article (*n* = 84) to determine its eligibility for our study according to the inclusion and exclusion criteria. CB and PB then compared their results to reach an agreement about which articles they recommended to include or exclude. After concluding this process, a total of 14 papers met the inclusion criteria and were included in our study.

### Charting the data

We charted and sorted data according to key themes and issues. Then we charted the extent, nature, and distribution of the included articles over various domains including: design, participants, technology, outcomes/objectives of the study, evaluation method/measures used, the geographical location of the study, data collection timeframe, key results and limitations (see ‘Table 2: Data Extraction’). Data extraction was conducted by CB and 20% was double-checked by PB. It was during the charting step that we began to realise our inability to answer our second research question as only three studies had used pedagogical concepts and theories to underpin their methodological design.

**Table 2:** Data Extraction Table

Authors and Date	Study Reviewed	Design	Participants	Technology	Objectives of the study	Evaluation method/measures used	The geographical location of the study	Data collection timeframe	Key results	Limitations
1.Yoganathan et al., 2018 [58]	360 degrees virtual reality video for the acquisition of knot tying skills: A randomised controlled trial	Pilot RCT	*N* = 40 male and female foundation year doctors. Participants were randomised to either the 360° video (n = 20) or 2D video teaching (*n* = 20).	Two identical videos demonstrating “a single-handed surgical reef knot” were produced based on Royal College of Surgeons (RCP) basic Surgical Skills video. Conventional video: recorded using an iPhone 7 (Apple) to play in HD 2D format on a laptop screen. 360° video: recorded using Insta360™ Nano and played through an HMD	To determine the effectiveness of knot tying skills taught with a 360° Virtual Reality video compared to 2-D video teaching.	Participants were shown their allocated video for 15 min; then, their ability to tie “a single-handed reef knot” was then assessed against a marking criterion developed by RCP.	UK	During and post.	Knot tying scores improved significantly in the 360° video teaching arm when compared with conventional methods (*p* = 0.04). More people in the 360° arm constructed a reef knot than in the 2D arm following face to face teaching (17/20 vs 12/20). This study shows there is significant use for 360° video technology in surgical training, both as a unique teaching aid and when used as an adjunct to conventional teaching.	The participants were not re-assessed to see if the difference in skill retention between the two arms was maintained.
2.Ulrich et al., 2019 [59]	Learning effectiveness of 360° video: experiences from a controlled experiment in healthcare education	Experimental study	*N* = 81 male and female physiotherapy students. Group 1 (*n* = 28) used 360° video. Group 2 (n = 26) used a regular video. Group 3 (*n* = 27) received traditional teaching.	The first group used 360° video shown on a VR-HMD (Samsung Gear VR). The second group used regular video shown on a laptop. All videos were recorded with the same teacher in physiotherapy education and the videos were recorded without using cut scenes to avoid interference from post-production (recording technology was not described).	To measure the effectiveness of 360° video in the students’ performance, satisfaction, and learning climate in an educational healthcare setting	A 3 × 3 factorial design. The 3 treatment groups were the independent variables (1) academic performance (2) perceived user satisfaction and (3) perception of learning climate. User satisfaction and perception of learning climate was measured using an adapted version of Chou and Liu’s [60] Learning Satisfaction Questionnaire.	Denmark	Pre and Post	360° video and regular video were less effective than conventional teaching in students’ learning satisfaction. 360° video was more effective at providing a “learning climate” (emotional impact). Results showed that traditional teaching was on most constructs as or more effective.	Small sample therefore, the sample may lack statistical power and generalising the results should be done with caution
3.Sultan et al. 2019 [61]	An Experimental Study on Usefulness of Virtual Reality 360 degree In Undergraduate Medical Education	Experimental study	*N* = 169 male and female undergraduate medical students. Group one (*N* = 57) experienced 360° video whereas group two (*N* = 112, control group) was provided with the interactive lecture	360° cameras, a smartphone and VR goggles (HMDs). Specific technology was not described. Group one received 3 VR videos which include (effective communication with patient, effective communication with relatives and non-health care professions and importance of the multidisciplinary team in healthcare).	To determine knowledge retention, perception level, satisfaction level and skill acquisition using 360 videos in medical education.	Objective Structured Clinical Examination (OSCE) and MCQ scores were used for measurement	Saudi Arabia	Pre and post.	The majority of students (93%) thought that 360° videoing could be used in medical education. Post-MCQs score was significantly higher in the 360° group when compared to the conventional group (*p* < 0.001). The OSCE score was also significant with the 360° group (*p*-< 0.001). 73% of participants were satisfied with the 360° video experience.	There were significantly fewer females when compared to males. Also all participants were UG medical students, the study’s strength would be increased by involving students from different academic levels.
4. Harrington et al., 2018 [62]	360° Operative Videos: A Randomised Cross-Over Study Evaluating Attentiveness and Information Retention	A randomised cross-over study	N = 40 male and female students in preclinical years of a medical university. Group 1: used the 360° video followed by the 2D experience Group 2 was reversed.	GoPro Omni was suspended and recorded an elective laparoscopic cholecystectomy. Samsung Gear VR HMDs enabled viewing followed by the 2D experience on a 75-in. television. Group 2 was reversed.	To evaluate variances of inattentiveness, information retention, and appraisal of 360° videos vs 2D formats.	8-point multiple choice questionnaires were used to assess engagement levels and task-unrelated images or thoughts. A 15-point questionnaire related to demographics and their video experiences.	Ireland	During (2 time-points) and post	During the 360° video participants had lower task-unrelated images or thoughts (*p* < 0.01) and were significantly engaged (*p* < 0.01) There were no significant variances in information retention between group 1 and 2. Most participants (65%) reported the 360° video as their learning platform of choice for learning, immersion, and entertainment.	Small participant numbers. The novelty factor of the new technology may have positively influence results. The retention questionnaires were more technically than observationally orientated
5. Huber et al., 2017 [63]	New dimensions in surgical training: immersive virtual reality laparoscopic simulation exhilarates surgical staff.	Feasibility study	N = 10 staff from the surgical department	The VR laparoscopic simulator was a LapSim, Samsung Gear 360° was used to record a video (including audio) sequence inside the operating room during laparoscopic surgery (artificial scenario). Startech online was used to transfer the video output signal of the simulator to a VR-ready laptop computer. Unity3D® was used to integrate the simulator display into the recorded 360° OR-video. Noise-canceling Bluetooth headphones (Bose® Quietcomfort® 35) were also used.	Assess and analyse the feasibility of using an immersive 360 video laparoscopy setup to measure extent of performance immersion and motion sickness.	The Validated Motion Sickness Scale [64] was used during the IVR session. Measurement of Presence and Its Consequences in Virtual Environments [65], was used after the course to evaluate the immersive effects the training.	Germany	During and post	Fine dissection took significantly longer for participants in the 360 video session [*p* = 0.022) with higher error rates. Motion sickness did not occur at any time for any participant. Participants in the 360 video session experienced immersion and a high level of exhilaration, “rarely thought about others in the room”, and had a strong feeling of presence.	The particularly small number of participants and the non-randomised study design.
6. Taubert et al., 2019 [66]	Virtual reality videos used in undergraduate palliative and oncology medical teaching: results of a pilot study.	Pilot Study	*N* = 72 male and female medical students	Oculus Rift HMD was used, videos were based on nausea and vomiting management in palliative care and oncology settings. They were also able to view the Radiotherapy Patient view VR experience. Details of camera used to video the experience was not available.	To determine the usefulness of using 360 videos as a teaching tool for undergraduate palliative and oncology medical teaching.	A questionnaire was used to measure comfort and ability to concentrate after the intervention. Participants were asked if they felt that the 360° experience suited their learning style and whether they would recommend.	UK	Post	Participants rated their experience on a scale of 0 to 10 (0 = worst, 10 = best). Their ability to concentrate had an av. score 8.44, suited their learning style av. score 8.31. 70 (97%) students confirmed that they would recommend this form of learning to a colleague.70 (97%) participants found the experience comfortable.	Validated measures measurement and analysis would have given more validity to the findings.
7.Pulijala et al., 2018 [67]	An innovative virtual reality training tool for orthognathic surgery.	Exploratory study	N = 7 consultant oral and maxillofacial surgeons	Stereoscopic visualisation of orthognathic surgery and 3D interaction using the Oculus Rift Development Kit 2, HMD and a Leap Motion controller. Six GoPro Hero cameras were used in the operating room	To determine the realism and usability, and the applicability of 360 videos for orthognathic surgical training.	Pre-intervention questionnaire regarding training needs 2. Post-intervention to gather data on the efficacy, usability, and acceptability of the system.	UK	Pre and post	Participant agreed with the validity of the content of the 360° video clips (mean score = 4.28). The participants also saw significant benefits from using the various components of the application (mean score = 4.46). Overall, there was validity for the content and use of the application of 360° videos to enable trainees to participate in a surgery environment	The particularly small number of participants and given that the technology was developed and evaluated by expert surgeons makes generalising the results difficult.
8. Buchman and Henderson, 2019 [68]	Qualitative Study of Interprofessional Communication through Immersive Virtual Reality 360 Video among Healthcare Students	Exploratory study	*N* = 39 male and female Inter-Professional (IP) healthcare students’	The VR 360 immersion lasted for 7 minutes and had six mini scenarios that merged into the one simulation VR 360 experience to immerse students in the role of a patient with macular degeneration and high-frequency hearing loss. Students were asked to wear a hearing headset and visual ocular set (no description of camera or HMD) experience.	To examine the experiences and attitudes of students following a 360° video which simulated the experience of a patient.	Immediately following each VR 360° video immersive experience, a 60 min focus group was held (semi-structured format).	USA	Post	Students found that 360 video experience increased their empathy and ability to understand a patient with hearing loss through verbal and nonverbal signs will lead to improved patient experience. The immersive element was evident in the comments about the realism and presence of feeling as if they “were in the patient’s shoes “or actively the patient’ in the 360° video experience.	Background noise was noted as a distraction. There was not an equal number of participants across healthcare professions.
9. Bernard, et al., (2019) [69]	Toward the development of 3-dimensional virtual reality video tutorials in the French neurosurgical residency program. Example of the combined petrosal approach in the French College of Neurosurgery.	Exploratory study	*N* = 17 seventeen senior neurosurgery residents and N = 5 ear, nose and throat (ENT) specialists	The study reports on the development of a commented 3D video displayed via Samsung Gear VR Virtual Reality Headset as a surgical tutorial. The team also developed 3D video capture, 3D legend and studio sound recording tools, to produce a 3D video adapted to several platforms	To to present a project of surgical neuro-anatomy teaching through a 3D video tutorial and assess the effectiveness of this teaching method.	At the end of the 3D surgical anatomy session students filled out an assessment form based on a 5-point Likert scale to assess the teaching and the positive and negative points of the teaching tool.	France	Post	Most participants had positive feelings about ease of use and their experience of the 3D video tutorial (n = 14, 63.6%) and 20 (90.9%) enjoyed using the video. 18 felt that the 3D video enhanced their understanding of the surgical approach (81.8%). 15 (68.2%) thought the video provided good 3D visualization of anatomical structures and 20 (90.9%) that it enabled better understanding of anatomical relationships. 12 (54.5%) considered that the cadaver dissection workshop was more instructive.	Sample size was small, study was specifically centered on the French training in neurosurgery in a specialist and complex area which requires anatomical knowledge.
10. Dawson et al., 2019 [70]	The use of virtual reality for public health education concerning Syrian refugee camps	Exploratory study	N = 17 public health students. N = 6 male and female first-year UG Public Health students from the UK and N = 11 s-year Public Health UG students from Lebanon	Samsung Gear 2 k was used by students in Lebanon to film footage of the visit for UK students which was edited into a 5-min clips from the camp and inside a family home. Students started viewing the footage using VR Box smartphone headsets then moved on to 2.5D viewing using a laptop or tablet screen to explore the footage.	Assess the impact of 360° videoing as a method of increasing UK students understanding of public health challenges faced by Syrian refugees	A questionnaire was used before and after (after included additional questions on the use of 360° video). A video interview was used for analysis after the exchange.	UK and Lebanon	Pre and Post	All participants involved in the evaluation deemed the use of 360° video as a positive experience where learning was enhanced, and emotions impacted. Participants thought that the 360° video had the potential to become more widely used in education as an adjunct or stand-alone teaching method. However, all users complained of simulator sickness, which was thought to detract from the immersive experience and possibly reduce the emotional impact of the experience.	Difficulties in regards to processing requirements for downloading 360° footage.
11. Taylor and Layland, 2018 [71]	Comparison study of the use of 360-degree video and non-360-degree video simulation and cybersickness symptoms in undergraduate healthcare curricula	Exploratory study	*N* = 60 undergraduate healthcare students participated in one of four identical (but different simulation) learning outcome simulation events.	This study compared four standard simulation tools, 360° video (using Samsung Gear VR), high-fidelity manikin, video case study and standardised patient, and analysed the self-reported cybersickness symptoms.	Determine the level of cybersickness symptoms in simulation learning and teaching tools in 360° video, manikin, standardised patient and video case study	Simulator Sickness Questionnaire (SSQ) [72].	UK	Post	360° videos are no more likely to provoke cybersickness symptoms than the other simulation methods. 360° videos were reported as less fatiguing than other modes of simulation learning. The fatigue symptom of participants in the non-360° condition was significantly higher than those in the 360° condition (*p* = 0.001). No other significant effects between simulations were found.	Need to control photorealism aspect, image refresh rate or alteration to the field of view to more accurately assess which can impact cybersickness.
12. Chan et al., 2021 [73]	Impact of 360 degree vs 2D Videos on Engagement in Anatomy Education	Experimental study	N = 39 fourth-year medical students. Participants were randomised into two groups: Group A viewed the 360° videos using the Oculus Go VR headset while Group B viewed the 2D videos on a laptop.	Samsung 360° Round Stereoscopic 3D camera and a NeXT PC stitching computer (NeXT Inc., Fremont, California) and 64 GB Oculus Go Headset was used Group A viewed the 360° videos using the Oculus Go VR headset. Each group watched both a 4 minute demonstration and explanation of upper endoscopy as well as a four-minute demonstration and explanation of liver biopsy in the same order.	To examine if 360° videos can promote increased engagement over standard two-dimensional (2D) videos among medical students learning anatomy.	Pre video: background questions as baseline for knowledge and attitudes towards immersive technology During video: participants answered questions to gauge their engagement Post video: participants completed a survey.	USA	Pre, during (3 time-points) and post	Seven out of eight questions assessing engagement were rated significantly higher in the 360° video group as opposed to the 2D group (Q1-6 and Q8 *p* < 0.05; Q9 *p* = 0.16), including feeling more stimulated and involved in the lab experience. Although the 2D group reported more ease and confidence with using the video playback feature (*p* < 0.05), there was no statistical difference in regards to perceived ease of learning between the two groups (*p* < 0.45). The 360° video was also rated as more practical (*p* < 0.007) and interesting (*p* < 0.001) than 2D.	Did not focus on the effectiveness of the use of immersive technologies to enhance knowledge. Did not assess changes in knowledge retention.
13. Sullivan et al., 2021 [74]	The Use of Virtual Reality Echocardiography in Medical Education	Cross-sectional observational study.	*N* = 15 medical students and staff. N = 3 cardiac electrophysiologists and N = 12 paediatric trainees, this included cardiology fellows (*n* = 4), general paediatric registrars (*n* = 7), and a senior house officer (*n* = 1).	Garmin VIRB® 360 and a head-mount display was used to record live echocardiography exams in a pediatric population. An Oculus Go™ was used to view the 360° immersive/VR videos.	To assess the utility of a VR echocardiogram in teaching echocardiography to paediatric trainees vs. live demonstration.	Trainees responded to a written questionnaire afterwards which included a 5-point Likert scale based on the usefulness of the technology.	Ireland	Post	All participants reported that VR echocardiography was a useful teaching tool and 87% (*n* = 13) rated it as good or very good on a 5-point Likert scale 93% (*n* = 14) said that they would recommend VR echocardiography to others as a teaching modality. The HMD was preferred by 80% (*n* = 12) in comparison to the tripod mount viewpoints. In comparison to live demonstration, 67% of respondents (*n* = 10) reported that VR echocardiography was the same or better. When asked how VR echo compared to traditional video for teaching, 80% (*n* = 12) rated it as better or much better.	A larger number of participants and using validated measures for data collection would have given more validity to the findings.
14. Lanzieri et al., 2021 [75]	Virtual Reality: An Immersive Tool for Social Work Students to Interact with Community Environments	Pilot study	*N* = 30 first-year Masters in Social Work (MSW) Students	The 360 VR simulation takes place in the Lower East Side (LES) of Manhattan, New York. The 360 VR simulation was developed by an internal media design and development team who used a Nikon KeyMission™ 360 camera and standard tripod and stitched together using Adobe Premiere software and Wonda VRTM, was used to edit the footage, an actor provided a recorded voice-over audio. Google Daydream headsets were used.	To investigate whether a virtual reality simulation impact student learning of a community environment	Pre-survey: designed to determine if participants’ prior experience influenced their performance. Post-survey: contained 28 questions divided into three parts (Learning Experience, Technology Experience, General Attitudes).	USA	Pre and post	Students felt the virtual simulation was like a real world (M = 5.63, SD =1.33), focused (M = 5.93, SD = 1.05), and immersed (M = 5.73, SD = 1.17). They also reported the ability to locate objects in the environment with ease (M = 5.67, SD = 1.27). Overall, perceptions on immersion rate were high (M = 5.79, SD = 1.2). Responses also showed an even split between “leaning toward VR” and “real-world environment” with a mean of 2.9 (SD = 1.24). Reflective questions alongside the technology was also considered helpful to increasing the immersive experience.	An experimental design that randomises participants into more than two groups to compare learning design features or methods using validated methods would have strengthened the study.

### Collating, summarising, and reporting the results

When reporting the data in our study, it was imperative that we used a consistent, clear approach. Therefore, we linked the results to the four areas relating to the ARCS model [42–44]: attention, relevance, confidence and satisfaction. We then framed our discussion in response to our research questions.

### PRISMA

**Fig. 3:**
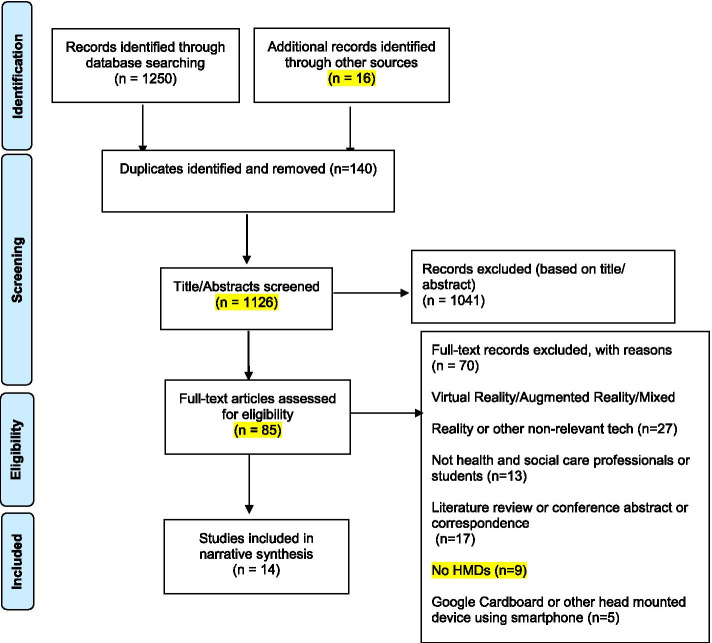
PRISMA Flow Diagram [33]

As noted, the Preferred Reporting Items for Systematic Reviews and Meta-Analyses (PRISMA) guidelines [56] was utilised (Fig. 3).

## Results

### Participant profile, study design, measurement, pedagogical concepts and theories

The collective number of participants are included in this review was *n* = 640 [58, 59, 61–63, 66–71, 73–75]**,** sample sizes ranged from *n* = 7-169. Studies generally did not provide an extensive profile of the research participants, such as information on gender or previous technology use. However, is was evident that almost the entire sample (92%, *n* = 616) were university students; eight of fourteen studies (*n* = 8/14) used a student cohort except for Bernard, et al. [69]; Pulijala et al. [67], Huber et al. [63] and Sullivan et al. [74]. Disciplines varied and samples included professionals and students training in medicine/surgery (*n* = 10; 71%) [58, 61–63, 66, 67, 69, 71, 73, 74], Public Health/ Health Care (*n* = 2; 14%) [68, 70], Physiotherapy (*n* = 1; 7%) [59] and Social work (*n* = 1; 7%) [75]. The majority of the studies (*n* = 5, 45%) were conducted in UK [58, 66, 67, 71, 69;], followed by USA (*n* = 3; 21%), Ireland (n = 2; 14%), Germany (*n* = 1; 7%), France (n = 1; 7%), Denmark (n = 1; 7%), and Saudi Arabia (*n* = 1; 7%).

### Study design

The 14 studies included a range of methodological designs. A randomised controlled trial (*n* = 1; 7%) [58], experimental studies (*n* = 3; 21%) [59, 61, 73], a randomised cross-over study (n = 1; 7%) [62]; cross-sectional observational study (n = 1; 7%) [74], a feasibility study (n = 1; 7%) [63], and a pilot study (n = 1; 14%) [66, 75]. The remaining studies (*n* = 5, 35%) used exploratory approaches [67–71].

### Measurement

Quantitative evaluation measures were the primary outcome measure in eight (*n* = 8; 56%) of the included studies followed by four that used mixed measures (*n* = 4; 28%) and two that used purely qualitative (*n* = 2; 14%). Skill enhancement was generally assessed through observation against marking criteria as specific to the task, e.g. Sultan et al. [61] used Objective Structured Clinical Examinations (OSCE) [76]. A range of validated questionnaires was used to gather data in relation to learning, Ulrich et al., [59] adapted Chou and Liu’s [60] Learning Satisfaction Questionnaire. Huber et al., [63] used The Validated Motion Sickness Scale [64], Measurement of Presence and Its Consequences in Virtual Environments [65] and Taylor and Layland [71] used The Simulator Sickness Questionnaire (SSQ) [72]. Other studies (*n* = 8; 56%) developed bespoke questionnaires using Likert Scale items to gather responses as relevant to their study, e.g. Taubert et al., [66] measured ability to concentrate when viewing the immersive 360° videos using a 0 to 10 (0 = worst, 10 = best) scale. Other studies were more focused on increasing knowledge measured through qualitative responses [68, 70] Qualitative data was gathered using several approaches, for example, Buchman and Henderson, [68] used post-intervention focus groups, whereas Dawson et al., [70] used semi-structured interviews pre and post-intervention. One study (7%) [70] interestingly used video recordings to capture and record session outcomes.

### Pedagogical concepts and theories

Learning theories were identifiable in three studies (*n* = 3; 21%) [59, 68, 75]. Ulrich et al. [59] introduces learning effectiveness theory [60] and focuses on three core factors – academic performance, learning satisfaction, and learning climate. Ulrich et al. [59] proposes three hypotheses to measure the learning effectiveness of immersive 360° videos through academic performance, learning satisfaction, and learning climate. Ulrich et al. [59] indicated that academic performance was assessed how a pedagogical technology is effective by measuring the student’s performance compared to their performance in other situations without the technology. Learning satisfaction was assessed by how pedagogical technology is effective by identifying the student’s overall satisfaction compared to situations where the technology is absent [59]. The focus on learning climate demonstrated how pedagogical technology is effective by understanding the student’s emotional and communicative environment in the pedagogical situation compared to situations where the technology is absent [59]. Ulrich [59] argues that because immersive 360° videos have similar characteristics to regular video but also can include a higher level of presence similar to traditional teaching, comparative judgment (based on Grover et al., [77]) is used to measure the learning effectiveness of immersive 360° videos against regular video and traditional teaching. Buchman and Henderson’s [68] study was informed by The Interprofessional Education Collaborative (IPEC) Framework [78] and the Communication Accommodation Theory (CAT) [79]. The IPEC has four distinct categories for Interprofessional pedagogy: values/ethics; roles/responsibilities; Interprofessional communication and teams and teamwork. Buchman and Henderson’s [68] study focused on the interprofessional communication domain. The CAT has been used in health and social care studies to look at communication across members of different generations [79, 80]. Lanzieri et al.’s design [75] was grounded in situated learning theory [81, 82] which focused on the importance of context in learning, the need for students to build strong mental models and transferable knowledge to function in real clinical settings, and the role of prior knowledge in decision-making and problem solving [81, 82].

## Reported outcomes

Due to the design of the studies and heterogeneity within reported outcome measures, it was difficult to deduce whether the immersive 360° experience increased the participants’ ability to self-regulate their behaviour to achieve their goals (ARCS-V – *volition* [45]). However, four areas relating to the ARCS model [42–44] were evident: attention, relevance, confidence and satisfaction.

The majority of studies (*n* = 9; 63%) recorded improved engagement/attention when using immersive 360 video [58, 61–63, 66, 67, 70, 73, 75]. For example, Harrington et al., [62] found that those in the 360 video group were more engaged (*p* < 0.01) throughout the experience and had lower task-unrelated images or thoughts (i.e. distraction from the task) (*p* < 0.01). In the majority of the surgical studies (*n* = 4; 31%) positive outcomes were reported relating to their ability to focus on the task. Huber et al. [63] described participants experienced a high level of exhilaration and rarely thought about others in the room [63], similarly Yoganathan et al. [58] and Pulijala et al. [67] also reported high levels of presence. Correspondingly, Chan et al. [73], found that overall engagement remained higher when using the immersive 360° videos: seven out of the eight survey questions assessing these factors were rated higher than the group using 2D Videos. In the post-video survey, there were statistically significant differences (*p* < 0.05) between the two groups in particular in regards to engagement and stimulated learning which was significantly more positive for those using the immersive 360° videos [73]. In Lanzieri et al.’s [75] study students felt focused (M = 5.93, SD = 1.05) and immersed (M = 5.73, SD = 1.17), overall, their perceptions on immersion rate were high (M = 5.79, SD = 1.2). Notably, Lanzieri et al.’s [75] found that the use of reflective questions was also considered helpful to learning, and positively correlated with the simulation’s immersive feeling. Taubert et al., [66] comments that students reported significant learning outcomes which was potential related to the novelty of the technology “Might have been the novelty factor but I learnt more from this 20 min VR thing than I have from many lectures”. It is evident from the majority of these studies that an immersive 360° environment has an effect on the user’s emotional response to the learning climate which in turn positively affected their attention, engagement and motivation to learn [63, 68, 70, 73, 75].

All (*n* = 7; 49%) of the studies [58, 61, 63, 67–69, 74] which focused their studies specifically on relevance to skill improvement or practice recorded benefits when using immersive 360 video. There were reported differences in skills development with Yoganathan et al. [58] suggesting that knot tying scores were significantly better in the immersive 360° videos’ teaching arm in comparison to conventional teaching methods (*p* = 0.04). In combination with conventional face to face skills teaching this difference continued (*p* = 0.01). A larger amount of participants in the 360° arm were able to construct a complete reef knot experiment than in the 2D arm after face to face teaching (17/20 vs 12/20). In Burnard et al.’s study [69] 18 participants commented that the 3D video enhanced their understanding of the surgical approach (81.8%)and 20 (90.9%) that it enabled better understanding of anatomical relationships. For Pulijala et al. [67] participants agreed with the validity of the content of the immersive 360° clips (mean score = 4.28) and saw significant benefits from using the various components of the application (mean score = 4.46). Sultan et al., [61] Post-MCQs score (out of 20) was significantly higher in the group using 360° in comparison to the conventional group (*p* < 0.001). The OSCE score was also higher with the 360° groups (*p* < 0.001). Huber et al. [63] was the only study to report more error rates when using Immersive Virtual Reality (IVR) during the cholecystectomy task in particular. They also noted that participants’ times for fine dissection were significantly longer during the IVR session (*p* = 0.022). However, Huber et al., [63] also noted that the questionnaire results underline the high immersion of the custom IVR setup with high levels of presence in the generated world, exhilaration, and loss of attention to others in the room. An interesting point raised by Buchman and Henderson [68] was the ability for the student to feel that they were in the patient’s shoes [68] enabling the learner to empathise with the patient perspective which has particular relevance for preparation to practice. Sullivan et al., [74] reported that all 15 participants reported that VR echocardiography was a useful teaching tool and 87% (*n* = 13) rated it as good or very good on a 5-point Likert scale. The findings of almost all the studies which focused on skill development suggests that when immersive 360° videos are used to facilitate the prototyping of virtual reality environments (VRE), this has positive pedagogical relevance.

The majority of studies (*n* = 12; 84%) reported confidence in the effectiveness of the technology as a tool for learning [58, 61, 62, 66–71, 73–75]. Three studies (21%) focused significantly on the usability of the technology [63, 66, 71] the other studies (*n* = 9; 63%) focused more on confidence to continue using the technology or recommend the technology to others. For example, Bernard et al., [69] reported that most students had positive feelings about ease of use and their experience of the 3D video tutorial (*n* = 14, 63.6%) and 20 (90.9%) enjoyed using the video. Sullivan et al., [74] reported that 93% (n = 14) of the same group said that they would recommend VR echocardiography to others as a teaching modality. Taylor and Layland [71] suggest that 360° videos were shown to have less associated fatigue than other (more conventional) simulation exercises; fatigue symptom of participants in the non-360° condition was statistically significantly higher than those in the 360° conditions (Z = − 3.20, *p* = 0.001). Dawson et al., [70] reported that all users complained of simulator sickness, which was thought to detract from the immersive experience and possibly reduce the emotional impact of the experience. However, this may be due to using cheaper equipment (Samsung Gear 360°) which does not come with a stitching function as difficulties with the technology were reported [70]. Huber et al. [63] reported that motion sickness did not occur at any time for any participant, therefore would be confident to continue with use. In contrast, Taubert et al., [66] suggest that of the 70 medical students who participated two students found the experience uncomfortable (1 = headset too tight; 1 = blurry visuals) but did not see this as a factor which would deter future use. Sullivan et al., [74] the head-mounted view was preferred by 80% (*n* = 12) in comparison to the tripod mount viewpoints, which notes confidence in the use of the head mounted displays (HMDs). Pulijala et al., [67] found that concerning the validity of the content of the surgical video clips within the application, the ‘*m’* score was 4.28, showing strong agreement with the effectiveness of the technology. Similarly, the participants in Chan et al.’s, [73] study rated 360° videos as more practical (*p* < 0.007) and interesting (*p* < 0.001) than 2D. Given that the all of the studies which asked whether they would recommend the technology to others responded positively and most noted confidence in its use, would suggest that there would be confidence in its adoption as an adjunct or ‘stand-alone’ pedagogical tool.

High levels of satisfaction with immersive 360 video technology was reported in the majority of studies (*n* = 11; 77%) [61–63, 66–70, 73–75]. By quantitative measures, Sultan et al. [61] suggest that the overall rating of satisfaction in the 360° experience showed a mean of 7.26 of 10 and raises an interesting point regarding the interactivity of using 360° videoing which supports experiential learning-by-doing (The Oculus Rift Development Kit 2 (DK2) VR headset and a Leap Motion controller were used in this application). Taubert et al. [66] asked whether this format suited their learning style; the average score was 8.31 (range 6-10). Harrington et al., [62] *M* appraisal levels for the 360° platform were positive with *m* responses of > 8/10 for the platform for learning, immersion, and entertainment. In other studies satisfaction was reported through qualitative feedback [68, 70]. Only in the learning effectiveness of immersive 360° video: experiences from a controlled experiment in healthcare education’ [59] (*n* = 1; 9%) was it reported traditional teaching was more effective than immersive 360° videos in students’ learning satisfaction. However, while Ulrich et al., [59] found more significant learning benefits from traditional (2D video) approaches, participants noted that they had a more significant emotional response within 360° environments. In contrast, Sullivan et al., [74] found that when assessing the utility of a VR echocardiogram in teaching echocardiography in comparison to live demonstration 67% of respondents (*n* = 10) reported that VR echocardiography was the same or better. Sullivan et al., [74] also noted similar findings, as when participants were asked how VR echo compared to traditional video for teaching, 80% (*n* = 12) rated it as better or much better. Lanzieri et al., [75] asked questions about participant’s preference for the immersive 360° video experience versus visiting a real-world environment to complete the same learning activity - responses also showed an even split between “leaning toward VR” and “real-world environment” with a mean of 2.9 (SD = 1.24). It is notable however that, even though immersive 360° videos may provide a higher level of presence over regular video Ulrich et al.’s [59] comparative results differed. In Ulrich et al.’s study [59] traditional teaching was equally or more effective on most constructs (academic performance, perceived user satisfaction, and perception of learning climate). Notably it [59] did not contain any form of interactivity and was based on exposure alone. So although satisfaction was high for the large majority of studies, it is evident that interactivity could be linked to greater satisfaction and thus more positive learning outcomes.

## Discussion

### Is there any evidence that immersive 360° videos increase learning outcomes and motivation to learn in health and social care education?

All (*n* = 14; 100%) of the studies reported positive learning outcomes as a result of using immersive 360° video as a learning tool when aligned with the ARCS model [42–44]. A range of outcomes relating to motivational learning were evident, including increased attention [58, 61–63, 66, 67, 70, 73, 75], relevance to skill improvement or practice [58, 61, 63, 67–69, 74], increased confidence in the learning environment [58, 61, 62, 66–71, 73–75] and satisfaction [61–63, 66–70, 73–75]. Although the outcomes did not specifically speak to the participants ability to self-regulate their behaviour and achieve their learning goals (ARCS-V – *volition* [45]), the large majority of the studies included in this review confirm that immersive 360° videos positively affected their motivation to learn. Therefore, it could be deduced that when the student is placed in an environment which closely simulates the in-vivo work environment these platforms directly and effectively address the skills gap by providing immersive, hands-on training. For example, Dawson et al., [70] suggests that students were able to move beyond what they experienced through text-based scenarios which facilitated students’ learning about a real-world situation that they would not have been able to access through other means. Throughout the majority of studies it was evident that increased role expectation and enhanced and contextualised learning environment positively affected participants’ motivation to learn. Therefore, it is apparent that immersive 360° videos may help teachers to introduce a sense of realism and high fidelity within teaching that is difficult to replicate in a classroom setting – this, in turn, may help equip the workforce to learn how to manage more complex scenarios or presentations. These findings resonate with Qiao, et al.’s [26] results in a scoping review relating to interprofessional education as students noted that they gained valuable insight into mutual roles and believed that this experience would benefit their role as a health care team member. Therefore, these applications may prosper particularly well with more junior medical personnel or students as an aid to theatre induction and protocols for safe practices. Furthermore, it could be reasoned that immersive 360° videos may have utility in health and social care education in particular due to the emotional responses generated through immersion and a sense of presence experienced by participants [59, 68, 70]. Makransky and Lilleholt’s [16] study supports this view as they found that increased immersion and consequential presence and emotional impact experienced by students can lead to positive educational outcomes. This level of abstraction within the learning experience aligns with the *‘Multimedia Cone of Abstraction’* (MCoA) theory [41, 46, 47] as learning experiences became more embedded as learners engaged with others in applied settings. However it’s evident that more exploration is necessary to more precisely define immersive 360° videos’ potential as a pedagogical tool. Sneelson et al.’s review [25] corroborates this perspective and suggests that 360° VR video might be more appropriate for certain types of learning such as promoting empathy, reflection, or skill-based knowledge as opposed to factual or conceptual knowledge. However, from our review, it would seem that the adoption of learning theories and concepts in the design of studies using immersive 360° videos would be helpful to more accurately assess utility as a learning tool across multiple health and social care settings.

### What are the key pedagogical concepts and theories that inform this area of research?

The second research question was concerned with the key pedagogical concepts and theories that inform this area of research. Unfortunately, this remained somewhat limited with key pedagogical and theory driven training clearly identifiable in only three studies (*n* = 3; 21%) [59, 68, 75]. As noted, Ulrich et al. [59] introduce learning effectiveness theory [46]; Buchman and Henderson’s [68] study was informed by The Interprofessional Education Collaborative (IPEC) Framework [78] and the Communication Accommodation Theory (CAT) [79, 80] and Lanzieri et al.’s [75] design was grounded in Situated Learning Theory [81, 82]. Considering that the large majority of the studies did not refer to pedagogical concepts or theories it could be reasoned that the reported high levels of satisfaction may be related to the novelty of the technology and the uniqueness of the experience rather than the learning task itself. Therefore the reported satisfaction experienced by participants cannot be directly linked to positive learning outcomes. This hypothesis corresponds with Rupp et al.’s., [37] study which reported that feelings of presence led to less information recalled during a simulation, potentially indicating the novelty of VR experiences may overwhelm learners. This example may be relevant to Huber et al.’s, [63] study in that the novelty of the VR experience may have actually had a detrimental effect considering that the number of mistakes were higher, and motion metrics were worse in Immersive Virtual Reality (IVR). This example in particular supports the distraction hypothesis that has been previously investigated with non-IVR setups [83]. Nevertheless, despite this omission, it could be reasoned that by placing learners in an authentic, but simulated environment using immersive 360° videos may increase and supplement the traditional and often abstract experiences that occur in health and social care learning environments. This theory also fits with the ‘Multimedia Cone of Abstraction’ (MCoA) theory, as learning experiences are levelled within the MCoA via a process of abstraction, whereby the likes of immersive 360° videos are more likely to be effective for more learners [46]. It is also evidenced that features of immersive 360° videos were advantageous over static 2D visualisations for learning [48, 49]. Furthermore these findings align with Hamilton et al.’s, [27] systematic review based on IVR as a pedagogical tool in education which found that the methods used to evaluate learning outcomes are often inadequate and this may affect the interpretation of IVR’s utility. When considering the response to this research question it is evident that there is a need for greater consideration of pedagogical concepts and theories especially more rigor in methodological approach is essential to understanding the potential of immersive 360° videos as a pedagogical tool.

### What are the limitations of using immersive 360° videos within health and social education?

Despite the lack of key pedagogical concepts and theories it is evident that this novel video platform delivers engaging and immersive benefits to audiences and may appeal to modern learning styles. However, it was also evident that there were significant limitations. Given that viewing immersive 360° videos is a largely passive experience, this disconnect between the user’s movement in the real world with their lack of movement in the virtual world can lead to a lack of presence [84]. The type of technology used was also linked to this limitation, 3D 360° camera arrays such as the GoPro Odyssey may add further perspective but are significantly more expensive, which may have been a significant barrier due to cost. Some studies did highlight some technical issues with the cheaper equipment such as the Samsung Gear 360°, which did not come with a stitching function [66, 70]. Furthermore, with some of the cheaper HDMs limitations included background noise and ocular discomfort that some participants reported as being a distraction [66]. Despite these difficulties, students generally experienced the immersive 360° videos as realistic and generally communicated satisfaction in usability. Although generally immersive 360° videos were deemed to provide a higher level of presence over regular video in Ulrich et al.’s study [59] outcomes were negative as traditional teaching was equally or more effective the immersive 360 videos on most constructs (academic performance, perceived user satisfaction, and perception of learning climate). This may have been related to the fact that it did not include any form interactivity and was based on exposure alone, however it is notable that Ulrich et al.’s study [59] was one of the few studies which actually used a learning theory to underpin their study. Although the majority of the participants in Burnard et al.’s study [69] reflected positively on their experience; over half (54.5%) commented that training with 360° videos cannot replace cadaver dissection courses, therefore appears necessary to integrate this training program in a complementary way. Interestingly, Lanzieri et al.’s [75] reflective questions was also considered helpful to learning, and positively correlated with the simulation’s immersive feeling, which raises further support associated with the need for more interactivity to fully experience immersion. It is evident that interactivity in an immersive environment leads to better learning outcomes [84], however from the studies included in this review, it is not clear what level of interactivity or user control is required as only one study [61], used technology (the Oculus Rift Development Kit 2 (DK2) and a Leap Motion controller) which facilitated this. Therefore, from this review the main limitations for the majority of studies [58, 59, 62, 63, 66–71, 73, 74] when using the 360° technology was that the locations of viewers were fixed. Therefore viewers have limited access to the angles captured by the cameras, and they cannot interact with the environment. This finding is consistent with previous research, demonstrating that using video for pedagogical purposes requires a high level of interactivity to be effective [85]. Nevertheless, as supported by Barreda-Ángeles, Aleix-Guillaume and Pereda-Baños [17] there is evidence to suggest that, even if interactivity is lacking, 360°video recreations of virtual environments still lead to realistic reactions on users. To this end, we suggest that the quality of the technology and the lack of interactivity were significant limitations. Given that despite these factors, user satisfaction remained high, therefore we hypothesise the novelty factor could increase curiosity and thus user satisfaction and further research is essential to uncover the specific impact on learning outcomes.

## Limitations

The main limitation of this review are the quality of the included studies. The majority of the studies used a small number of participants (lowest *n* = 7); therefore, the samples lack statistical power and generalising the results should be done with caution. The majority also noted the non-randomised study design (*n* = 4 were randomised) and the lack of reassessment post the 360° experience (*n* = 0 re-assessed post the 360° experience). The lack of robust experimental design would suggest that the evidence to date is weak and in need of strengthening. Another limitation of the studies was that they often used a convenience sample (*n* = 14; 100%); therefore the participants were not evenly distributed among the various health and social care professions or across disciplines or at various stages of their careers. A final limitation to note was, to control the influx of new dependent variables, we focused this review on health and social care education rather than including a range of educational areas with different opposing objectives.

## Future directions

A variety of immersive 360° video applications have been evaluated in various fields of study, predominantly medical pedagogy with a particular focus of surgical training. However psychology, applied social sciences and mental health pedagogy remain underexplored, which points towards a need for future studies in these areas. Three points are of note: further robust studies to test the learning effectiveness of the technology, affordability in technology for global application and further interactivity. Although it is evident that immersive 360° videos have a positive effect on the user’s emotional response to the learning climate, which has a significant effect on users’ motivation to learn, it was not clear what impact immersion had of learning outcomes in the included studies which were based on health and social care education. Notably a recent study investigated the feasibility and efficacy of a self-administered Mobile Assisted Language Learning homework training based on immersive 360° videos and found that the immersion was the key factor determining greater English learning outcomes [86]. Therefore, future work in health and social care education should also explore the learning outcomes associated with immersion in health and social care education [40], affective content and the impact on presence [39]. As Baños et al.’s [87] study suggests that immersion was more relevant for non-emotional environments than for emotional ones, this is a point of particular relevance in preparation to practice for health and social care. Improved navigation may increase a learner’s sense of presence in the virtual learning environment, which, in return, may enhance the learning experience compared to regular video [29, 88]. Unfortunately, studies (to date) do not generally explore this as a practical option using 360° technology, despite this being a possibility. This may provide a viable opportunity for future studies exploring interactivity in immersive 360° video as a low-cost alternative to VR. These findings are corroborated by Kavanagh et al. ‘s [32, 89] work as they also suggest future work should investigate ways to direct user attention during 360° lectures, either through overlaying information directly onto the video or by developing an alternative to the standard Oculus Video player with increased functionalities. Nevertheless, the overall findings suggest that immersive 360° videos are generally accessible and a financially feasible alternative to VR for use in health and social care as a pedagogical tool for independent teaching and also when used as an adjunct to conventional face to face teaching.

## Conclusion

Evidence would suggest that the use of 360° technology as a pedagogical tool is a viable alternative to face-to-face teaching to support pedagogy and training which offers portable, on-demand training that can be used anytime, anywhere. 360° videos have the potential to generate experiences which induce similar emotional and cognitive reactions to real-life situations and thus could potentially provide more realistic role expectation [31–34]. It is also evident that immersive 360° learning environments may also serve to demonstrate more complex, or novel approaches within health and social care practice, give learners the opportunity to experience failure in a ‘safe setting’ and the opportunity to engage with sensitive and potentially difficult situations. With a strained health and social care system and a pending health and social care staff shortage, training through 360° videos may contribute positively to addressing the need for rapid Health and Social Care innovation. Despite this evidence, current research using immersive 360° videos in health and social care pedagogy has primarily focused on effectiveness and efficacy research, with few studies evaluating “scaling up” or implementing such interventions in larger populations. Therefore the efficacy and effectiveness of such interventions are yet to be fully realised due to small sample sizes, lack of randomised control trials, and a gap in reporting key intervention qualities and outcomes. Nevertheless, it is evident that the use of immersive 360° videos may be particularly relevant given the urgent requirement for rapid pedagogical development given the recent COVID-19 outbreak [90]. Future studies would do well provide detailed information (including motivations, learning process and learning outcomes for the learners) on the learning theories that has informed the development of immersive 360° applications for health and social care training. In conclusion, by using 360° technology, students can be immersed in health, and social care situations that infrequently occur or are difficult to access in the real world. The evident advantages of this technology and the evidence of positive learning outcomes in the included studies may signal a new generation of approaches training health and social care training.

## Data Availability

All data generated or analysed during this review are included in this article [and its supplementary files].

## Abbreviations

AR: Augmented Reality
CAT: Communication Accommodation Theory
CoE: Cone of Experience
HMDs: Head-Mounted Displays
HEE: Health Education England
IVR: Immersive Virtual Reality
IPEC: Interprofessional Education Collaborative
DK2: Oculus Rift Development Kit 2
PRISMA: Preferred Reporting Items for Systematic Reviews and Meta-Analyses
MCoA: Multimedia Cone of Abstraction
SoP: Sense of Presence
3-D: Three-dimensional
2D: Two dimensional
VR: Virtual Reality
VRE: Virtual reality environments
